# A novel fractional model for the projection of households using wealth index quintiles

**DOI:** 10.1371/journal.pone.0277472

**Published:** 2022-11-17

**Authors:** Shakoor Ahmad, Shumaila Javeed, Saqlain Raza, Dumitru Baleanu

**Affiliations:** 1 Department of Mathematics, COMSATS University Islamabad, Chak Shahzad Islamabad, Pakistan; 2 Department of Mathematics, Near East University, Mathematics Research Center, Nicosia /Mersin, Turkey; 3 Respiratory Care Department, College of Applied Medical Sciences in Jubail, Imam Abdulrahman bin Faisal University, Jubail, Saudi Arabia; 4 Department of Mathematics, Cankaya University, Ankara, Turkey; 5 Institute of Space Sciences, Magurele-Bucharest, Romania; Jinan University, China, HONG KONG

## Abstract

Forecasting household assets provides a better opportunity to plan their socioeconomic activities for the future. Fractional mathematical models offer to model the asset-holding data into a piece of scientific evidence in addition to forecasting their future value. This research focuses on the development of a new fractional mathematical model based on the wealth index quintile (WIQ) data. To accomplish the objective, we used the system of coupled fractional differential equations by defining the fractional term with the Caputo derivative and verified it with the stability tests considering the steady-state solution. A numerical solution of the model was obtained using the Adams-Bashforth-Moulton method. To validate the model, we used real-time data obtained from the household series of surveys in Punjab, Pakistan. Different case studies that elucidate the effect of quintiles on the population are also presented. The accuracy of results between real-world and simulated data was compared using absolute and relative errors. The synchronization between the simulated results and real-time data verifies the formulation of the fractional WIQ model. This fractional model can be utilized to predict the approximation of the asset-holding of the households. Due to its relative nature, the model also provides the opportunity for the researchers to use the WIQs of their respective regions to forecast the households’ socioeconomic conditions.

## Introduction

A mathematical model is a synopsis of a system of equations developed using mathematical concepts and relevant data sets such as, physical [1], biological [2], or economic data [3], in order to obtain insights and, to prepare for the predicted values obtained from the model [4]. It is an interesting tool to formulate real-world problems, which are useful for evaluating, controlling, and forecasting the tendency of issues and diseases. However, a dynamic mathematical system helps in describing the time dependence of a point in the geometric space [5]. From the economic perspective, these models help in predicting the link between the households’ socioeconomic conditions & deprivations, fluctuations in their assets, economic development, financial growth, economic policies, and other relevant factors [6–10].

In recent years, fractional calculus (FC) has raised its importance in understanding the complex dynamics of real financial and economic systems. FC theory has not only grown quickly, but it has also found widespread application in a variety of fields [11–16]. In reality, the majority of economic mechanisms have long-term memories. Fractional derivatives are superior to integer derivatives for analyzing economic models because they are associated with the entire time domain of the economic system. Some long-memory continuous-time mathematical frameworks for illustrating economic dynamics have been developed [17–19]. Wang and Huang discovered some stability and chaos constraints using a delayed fractional-order financial technique [20]. Junhai Ma and Wenbo Ren designed a fractional-order macroeconomic system and identified some Hopf bifurcation and stability conditions [21]. Javeed *et* *al* presented the qualitative analysis of dynamical models for Dengue [22, 23].

Although the derivatives with integer-order have the property of locality, the integer-order differential equations used for data modeling and mathematical economics cannot describe the processes with memory or non-locality. The differential equation of the fractional-order is a mathematical model that can overcome the shortcomings of differential frameworks of the integer-order. The memory property of the fractional-order derivative is a significant benefit of using Fractional Calculus (FC) or transforming integer-order differential frameworks to non-integer order differential models [24]. This is very critical for economic models, which usually address both the past as well as the impact of the past and present on the future. Memory proficiency in fractional differential techniques is central to our motivation.

This work aimed to construct the fractional model named the factional Wealth Index Quintile (WIQ) model where WIQs have been used as variables and are a function of time *t*. The WIQ is an important measure in describing the asset-based inequalities between the rich and poor in the region. It is also a substantial source to examine the inequalities in the possession of items and assets, water and sanitation, access to healthcare services, and other aspects of the household wealth between rich and poor. However, the model has also the ability to forecast the dynamic sources of shift between the quintiles. We have formulated our model in terms of coupled fractional differential equations and solved them using the Adams-Bashforth-Moulton method. The results are compared with the real-time data obtained from the Multiple Indicator Cluster Survey (MICS) in Punjab Pakistan, from 2007 to 2018. The validity of the model has also been verified through stability analysis and discussed by several test problems and parametric studies, as well. This study highlights the importance of FC.

WIQ in different formats have been studied in association with depression [25], child mortality [26], obesity [27, 28] and child discipline [29]. Apart from the association of WIQs with some human biological and psychological characteristics, we rarely find any study that captures the movement of these indices for the future. Our study not only forecasts the WIQs but is also a source of controlling these characteristics that affect human socioeconomic and health badly.

The construction of the fractional WIQ model is based on the data collected from four episodes of the Multiple Indicator Cluster Survey (MICS) in Punjab Pakistan, from 2007 to 2018 (Table 1). These surveys are periodically conducted by the bureau of statistics Punjab in collaboration with UNICEF. The survey gives cross-nationally comparable information about the social health of women and children in addition to other indicators. A quintile is representative of 20% of the population, and, its construction is based on the assets of the household, their socioeconomic conditions, and access to mother and children healthcare services, etc [30]. It is an important component in determining the multidimensional poverty index [31].

**Table 1:** Numerical simulation for the year 2017 for *α* = 0.1, *α* = 0.3 and *α* = 0.5.

WIQ	Real	For *α* = 0.1			For *α* = 0.3			For *α* = 0.5		
		Simulated	AE	RE	Simulated	AE	RE	Simulated	AE	RE
*Q*_1_	19.991	18.755	1.236	0.062	18.594	1.397	0.070	18.386	1.605	0.080
*Q*_2_	20.009	19.807	0.202	0.010	19.881	0.128	0.006	19.965	0.044	0.002
*Q*_3_	19.994	20.348	0.354	0.018	20.444	0.450	0.023	20.578	0.584	0.029
*Q*_4_	19.995	21.326	1.331	0.067	21.390	1.395	0.070	21.479	1.484	0.074
*Q*_5_	20.011	19.764	0.247	0.012	19.691	0.320	0.012	19.592	0.419	0.021

## Wealth index quintiles: Introduction, construction, and importance

Wealth as a household characteristic ranks the socioeconomic status of households such as poor, middle, or rich. It is a necessary tool used to acquire assets, services, facilities, consumptions, and expenditures for a healthy life. Before the 1990s, a household’s socioeconomic status was interrogated using their incomes and expenditures. However, the data accuracy was a challenge that require extensive public money for household surveys. Besides, accurate income determination and expenditure indicators were poorly established especially in low-income countries. The existence of inequality was used to measure average variation across the population. Such an analysis provides only a partial picture of how different socioeconomic indicators are distributed across the population and cannot fully quantify the degree of socioeconomic inequality when the data is of continuous nature [32].

The wealth index is a quantitative measure of ranking households for their socioeconomic and health status. It is a composite measure of a household’s cumulative living standards. World Bank and Demographic and Health Survey (DHS) teams established the index to account for the socioeconomic and health status of households in a country or region on a continuous scale of relative wealth. Regional variations in hygiene, water and sanitation are common. Using wealth quintiles, it is possible to analyze regional inequalities in assets, services, and facilities between the rich and poor. WIQs have the potential to monitor the distribution of public resources to various groups of the population, nutrition & health indicators equally among all the ranks especially the poorest of the poor. The WIQ was initially developed from present information on household asset-holding and facilities to analyze health, population, education, and other sign of societal well-being according to economic rank. It is a proxy measure where real income and expenditure data are not available. The WIQ data was obtained from the Bureau of statistics, Punjab for the years 2007−2008, 2010−2011, 2014−2015, and 2017−2018.

Principal Component Analysis (PCA) technique [33] has been used to construct WIQs across developing countries. For this, we use the information on household assets including ownership of consumer goods, water and sanitation, socioeconomic conditions of households, and other relevant characteristics to generate weights. A wealth score is assigned to every household based on these weights and ranks them according to the wealth scores. Finally, all the households are divided into five equal parts called quintiles from poorest to richest.

Following [34] to avoid ‘clumping’ and ‘truncation’ problems and to minimize measurement error, the range of asset variables is broad enough in construction of PCA: number of rooms used for sleeping, floor material, roof material, walls material outside the house, cooking oil, household accessories (gas, electricity, television, radio, telephone, refrigerator, air conditioner, air cooler/fan, computer, washing machine/dryer, cooking range/microwave, iron, water filter, sewing machine, and donkey pump/turbine), utilities used by the individuals of the house (car, mobile, watch, bicycle, motorcycle/scooter, bus/truck, a boat with motor, tractor/trolley, animal drawn-cart), household/land ownership, having animals (chickens/ducks/turkey, sheep, goats, cattle, buffaloes, milk cows, donkeys, camels or mules, horses, and donkeys), bank account, sources of drinking water and toilet types. The wealth index will be supposed to take the prime lasting wealth from information on the household assets and is meant to provide a rank to the households through wealth, from lowest to highest. Further information on the wealth index construction can be found in [35–37].

## Mathematical model

In this section, we have formulated a linear mathematical model in terms of quintiles *Q*_1_, *Q*_2_, *Q*_3_, *Q*_4_ and *Q*_5_ where quintiles are a function of time. The linear model is chosen as the behavior of quintiles is linear. The parameters ‘*k*’, ‘*a*’, ‘*c*’, ‘*b*’, ‘*e*’, ‘*f*’, ‘*r*’, ‘*s*’ and ‘*h*’ are the transition rates of the households. The fractional WIQ model has been expressed in terms of coupled fractional differential equation:

$$\begin{array}{c} \frac{d^{\alpha }Q_{1}\left(t\right)}{dt^{\alpha }}=kQ_{2}\left(t\right)-aQ_{1}\left(t\right)+cQ_{2}\left(t\right), \end{array}$$ (1)

$$\begin{array}{c} \frac{d^{\alpha }Q_{2}\left(t\right)}{dt^{\alpha }}=kQ_{3}\left(t\right)-kQ_{2}\left(t\right)+aQ_{1}\left(t\right)-cQ_{2}\left(t\right)-bQ_{2}\left(t\right)+eQ_{3}\left(t\right), \end{array}$$ (2)

$$\begin{array}{c} \frac{d^{\alpha }Q_{3}\left(t\right)}{dt^{\alpha }}=kQ_{4}\left(t\right)-kQ_{3}\left(t\right)+bQ_{2}\left(t\right)-eQ_{3}\left(t\right)-fQ_{3}\left(t\right)+rQ_{4}\left(t\right), \end{array}$$ (3)

$$\begin{array}{c} \frac{d^{\alpha }Q_{4}\left(t\right)}{dt^{\alpha }}=kQ_{5}\left(t\right)-kQ_{4}\left(t\right)+fQ_{3}\left(t\right)-rQ_{4}\left(t\right)-sQ_{4}\left(t\right)+hQ{}_{5}\left(t\right), \end{array}$$ (4)

$$\begin{array}{c} \frac{d^{\alpha }Q_{5}\left(t\right)}{dt^{\alpha }}=-kQ_{5}\left(t\right)+sQ_{4}\left(t\right)-hQ_{5}\left(t\right), \end{array}$$ (5)

with initial conditions *Q*_1_(*t* = 0) = *β*_1_, *Q*_2_(*t* = 0) = *β*_2_, *Q*_3_(*t* = 0) = *β*_3_, *Q*_4_(*t* = 0) = *β*_4_ and *Q*_5_(*t* = 0) = *β*_5_; where *α* is the order of the derivative. This system of equations shows the transition of quintiles in terms of parameters. These parameters illustrate the upper or lower socioeconomic position of the quintiles. The parameters ‘*a*’, ‘*b*’, ‘*f*’and ‘*s*’are related to the spontaneous financial advantage of those households who have raised the ranking from lower to upper subsequent quintile, respectively. Similarly the parameters ‘*c*’, ‘*e*’, ‘*r*’and ‘*h*’are related to financial loss of those households who declined their quintile ranking in subsequent manner, respectively. The parameter ‘*k*’denotes the possibility of households leading to the inferior level due to any reason. All the transition rates ‘*k*’, ‘*a*’, ‘*c*’, ‘*b*’, ‘*e*’, ‘*f*’, ‘*r*’, ‘*s*’and ‘*h*’ are positive numbers.

Notice that

$$\begin{array}{c} \frac{d^{\alpha }Q_{1}\left(t\right)}{dt^{\alpha }}+\frac{d^{\alpha }Q_{2}\left(t\right)}{dt^{\alpha }}+\frac{d^{\alpha }Q_{3}\left(t\right)}{dt^{\alpha }}+\frac{d^{\alpha }Q_{4}\left(t\right)}{dt^{\alpha }}+\frac{d^{\alpha }Q_{5}\left(t\right)}{dt^{\alpha }}=0. \end{array}$$ (6)

Total number of households remains constant, *i.e*, *Q*_1_ + *Q*_2_ + *Q*_3_ + *Q*_4_ + *Q*_5_ = *N*.

The Caputo-derivative can be defined as:

$$\begin{array}{c} {}^{C}D_{t}^{\alpha }Q\left(t\right)≔\frac{1}{\Gamma (n-\alpha )}\int_{0}^{t}\frac{Q^{n}\left(\tau \right)}{{\left(t-\tau \right)}^{\alpha -n+1}}d\tau . \end{array}$$ (7)

We use the Caputo derivative, but the other fractional derivatives e.g. Riemann–Liouville derivative, and Caputo-Fabrizio derivative can also be used [38]. The Caputo fractional derivative has the advantage of allowing conventional initial and boundary conditions to be included in the problem formulation. Furthermore, the Caputo fractional derivative for a constant is zero. The physical interpretation using the Caputo derivative is easy in real-life problems [39].

The following expressions are important to calculate the values of the parameters used for the numerical simulation of the model.

$$\begin{array}{c} a≃\frac{\Delta Q_{2}\left(t\right)Q_{1}\to \;Q_{2}}{Q_{1}\left(t\right)\Delta \left(t\right)}, \end{array}$$ (8)

here, $\frac{\Delta Q_{2}\left(t\right)Q_{1}\to \;Q_{2}}{\Delta t}$ mentioned that increase in the number of households per time step from *Q*_1_ to *Q*_2_ *i.e*, the household has now shifted to economic betterment than the poorest *Q*_1_ households.

$$\begin{array}{c} b≃\frac{\Delta Q_{3}\left(t\right)Q_{2}\to \;Q_{3}}{Q_{2}\left(t\right)\Delta \left(t\right)}, \end{array}$$ (9)

$\frac{\Delta Q_{3}\left(t\right)Q_{2}\to \;Q_{3}}{\Delta t}$ shows the addition of households in *Q*_3_ *i.e*, the households from *Q*_2_ have improved their economic position and jumped into *Q*_3_.

$$\begin{array}{c} f≃\frac{\Delta Q_{4}\left(t\right)Q_{3}\to \;Q_{4}}{Q_{3}\left(t\right)\Delta \left(t\right)}, \end{array}$$ (10)

$$\begin{array}{c} s≃\frac{\Delta Q_{5}\left(t\right)Q_{4}\to \;Q_{5}}{Q_{4}\left(t\right)\Delta \left(t\right)}. \end{array}$$ (11)

Similarly, *f* and *s* show the transition of households in the higher quintiles. Contrary to this, following Eqs (12–15) show the downward transition of households from their respective quintile in time *t* (excluding the transition related to the probability *k*).

$$\begin{array}{c} c≃\frac{\Delta Q_{1}\left(t\right)Q_{2}\to \;Q_{1}}{Q_{2}\left(t\right)\Delta \left(t\right)}, \end{array}$$ (12)

$$\begin{array}{c} e≃\frac{\Delta Q_{2}\left(t\right)Q_{3}\to \;Q_{2}}{Q_{3}\left(t\right)\Delta \left(t\right)}, \end{array}$$ (13)

$$\begin{array}{c} r≃\frac{\Delta Q_{3}\left(t\right)Q_{4}\to \;Q_{3}}{Q_{4}\left(t\right)\Delta \left(t\right)}, \end{array}$$ (14)

$$\begin{array}{c} h≃\frac{\Delta Q_{4}\left(t\right)Q_{5}\to \;Q_{4}}{Q_{5}\left(t\right)\Delta \left(t\right)}. \end{array}$$ (15)

A similar approach was already applied in an epidemiological model to estimate the epidemiological parameters [40, 41].

## Stability analysis of the fractional WIQ model

**Theorem.** The system is asymptotically stable if and only if all the eigenvalues of the matrix have negative real parts [42].

**Stationary solution.** The Caputo fractional derivative has the advantage of allowing conventional initial and boundary conditions to be included in the problem formulation. Furthermore, the Caputo fractional derivative for a constant is zero. The stationary solution $Q_{1}^{*}$, $Q_{2}^{*}$, $Q_{3}^{*}$, $Q_{4}^{*}$ and $Q_{5}^{*}$=*N*-$Q_{1}^{*}$-$Q_{2}^{*}$-$Q_{3}^{*}$-$Q_{4}^{*}$ (in which $Q_{1}^{*}$, $Q_{2}^{*}$, $Q_{3}^{*}$, $Q_{4}^{*}$, $Q_{5}^{*}$ are constants) satisfying

$$\begin{array}{c} \frac{d^{\alpha }Q_{1}\left(t\right)}{dt^{\alpha }}=0,\frac{d^{\alpha }Q_{2}\left(t\right)}{dt^{\alpha }}=0,\frac{d^{\alpha }Q_{3}\left(t\right)}{dt^{\alpha }}=0,\frac{d^{\alpha }Q_{4}\left(t\right)}{dt^{\alpha }}=0,\frac{d^{\alpha }Q_{5}\left(t\right)}{dt^{\alpha }}=0, \end{array}$$ (16)

for any time *t* of the above system of equation is given as:

$Q_{1}^{*}=\frac{RN}{k^{4}+Sk^{3}+Tk^{2}+Uk+V}$ ,

$Q_{2}^{*}=\frac{a}{k+c}Q_{1}^{*}$ ,

$Q_{3}^{*}=\frac{ab}{\left(k+e)(k+c\right)}Q_{1}^{*}$ ,

$Q_{4}^{*}=\frac{fab}{\left(k+r)(k+e)(k+c\right)}Q_{1}^{*}$ ,

where *R* = (*k* + *r*)(*e* + *k*)(*c* + *k*)(*h* + *k*), *S* = *a* + *e* + *c* + *h* + *r*, *T* = *ab* + *ae* + *ah* + *ar* + *ch* + *ec* + *eh* + *er* + *cr* + *hr*, *U* = *abf* + *abr* + *chr* + *ehr* + *hec* + *ecr* + *aeh* + *rae* + *har* + *abh* and *V* = *cehr* + *hare* + *abhr* + *sfab* + *hfab*. The stability analysis of the steady state solution is obtained by writing the first four fractional differential equations with *Q*_5_(*t*) = *N* − *Q*_1_(*t*) − *Q*_2_(*t*) − *Q*_3_(*t*) − *Q*_4_(*t*). We linearize the system of equations,

$$\begin{array}{c} \frac{d^{\alpha }Q_{1}\left(t\right)}{dt^{\alpha }}=kQ_{2}-aQ_{1}+cQ_{2}, \end{array}$$ (17)

$$\begin{array}{c} \frac{d^{\alpha }Q_{2}\left(t\right)}{dt^{\alpha }}=kQ_{3}-kQ_{2}+aQ_{1}-cQ_{2}-bQ_{2}+eQ_{3}, \end{array}$$ (18)

$$\begin{array}{c} \frac{d^{\alpha }Q_{3}\left(t\right)}{dt^{\alpha }}=kQ_{4}-kQ_{3}+bQ_{2}-eQ_{3}-fQ_{3}+rQ_{4}, \end{array}$$ (19)

$$\begin{array}{c} \frac{d^{\alpha }Q_{4}\left(t\right)}{dt^{\alpha }}=kQ_{5}-kQ_{4}+fQ_{3}-rQ_{4}-sQ_{4}+hQ{}_{5}. \end{array}$$ (20)

Solving above Eqs (17–20), we get the following matrix:

$$\begin{array}{c} \left[\begin{array}{cccc} -2k-s-r-h & -k+f-h & -k-h & -k-h\\ k+r & -k-f-e & b & 0\\ 0 & k+e & -k-c-b & a\\ 0 & 0 & k+c & -a \end{array}\right] \end{array}$$ (21)

Using MATLAB, the matrix (c.f. Eq 21 leads to the following characteristics equation:

$$\lambda^{4}+(a+b+c+e+f+h+4k+r+s)\lambda^{3}+(ab+ae+ce+af+bf+cf+ah+bh+ch+eh+fh+3ak+2bk+3ck+3ek+2fk+3hk+6k^{2}+ar+br+cr+er+hr+3kr+as+bs+cs+es+fs+2ks)\lambda^{2}+(abf+abh+aeh+ceh+afh+bfh+cfh+2abk+2aek+2cek+afk+bfk+cfk+2ahk+bhk+2chk+2ehk+fhk+3ak^{2}+bk^{2}+3ck^{2}+3ek^{2}+fk^{2}+3hk^{2}+4k^{3}+abr+aer+cer+ahr+bhr+chr+ehr+2akr+bkr+2ckr+2ekr+2hkr+3k^{2}r+abs+aes+ces+afs+bfs+cfs+aks+cks+eks+fks+k^{2}s)\lambda +(abfh+abfk+abhk+aehk+cehk+abk^{2}+aek^{2}+cek^{2}+ahk^{2}+chk^{2}+ehk^{2}+ak^{3}+ck^{3}+ek^{3}+hk^{3}+k^{4}+abhr+aehr+cehr+abkr+aekr+cekr+ahkr+chkr+ehkr+ak^{2}r+ck^{2}r+ek^{2}r+hk^{2}r+k^{3}r+abfs)=0$$

The polynomial λ^4^ + *A*λ^3^ + *B*λ^2^ + *C*λ + *E* = 0, shows that if *A* > 0, *B* > 0, *C* > 0 and *E* > 0 have negative eigenvalues which is the case here. This steady-state solution is asymptotically stable as the given eigenvalues have negative real parts [43, 44].

By using the expressions given in Eqs (8–15), we can find the values of parameters as follows:

*a* ≃ 0.11,
*b* ≃ 0.08,
*f* ≃ 0.07,
*s* ≃ 0.05,
*c* ≃ 0.09,
*e* ≃ 0.07,
*r* ≃ 0.06,
*h* ≃ 0.05.

Now using these values of parameters and *k* = 0.005 in matrix (c.f. Eq 21, then the eigenvalues of the matrix will be λ_1_ = −0.2758153121, λ_2_ = −0.1812260272, λ_3_ = −0.0944116031 & λ_4_ = −0.0292164791, which concludes the asymptotic stability of the system of equations for the steady-state solutions of the model.

The Adams-Bashforth-Moulton method is employed to numerically simulate the fractional WIQ model.

## Numerical method

The system Eqs (1–5) has the following form,

$$\begin{array}{c} D_{t_{0}}^{\alpha }=f\left(t,y\left(t\right)\right),y\left(t_{0}\right)=y_{0}. \end{array}$$ (22)

After applying the fractional operator on the differential equation with the initial conditions, so converting the equation into the consequent equation

$$\begin{array}{c} y\left(t\right)=y_{0}+\frac{1}{\Gamma (\alpha )}\int_{t_{0}}^{t}{\left(t-u\right)}^{\alpha -1}f\left(u,y\left(u\right)\right)du, \end{array}$$ (23)

Eq (23) is called a Volterra equation of the second kind. Adams-Bashforth Moulton technique is used for first-order equations, therefore we choose for both integrators [45]. In order to derive this technique, the key is to transform the original fractional differential Eq (22) into the equivalent singular Volterra Eq (23). We apply the product trapezoidal quadrature formula with nodes *t*_*j*_(*j* = 0, 1…, *n* + 1), taken with respect to the weight function (*t*_*n*+1_ − *u*)^*α*−1^, to replace the integral. In simple words, we apply approximation as follows,

$$\begin{array}{c} \int_{t_{0}}^{t_{n+1}}{\left(t_{n+1}-u\right)}^{\alpha -1}g\left(u\right)\equiv \int_{t_{0}}^{t_{n}+1}{\left(t_{n+1}-u\right)}^{\alpha -1}g_{n+1}\left(u\right)du, \end{array}$$ (24)

where *g*_*n*+1_ is the piecewise linear interpolant for *g* whose nodes and knots are selected at the *t*_*j*_(*j* = 0, 1…, *n* + 1).

By applying the product rectangle rule we replace the integral on the right-hand side of Eq (24), so

$$\begin{array}{c} \frac{t_{0}}{t_{n}+1}{\left(t_{n+1}-u\right)}^{\alpha -1}g\left(u\right)du\equiv \sum_{j=0}^{n+1}b_{j,n+1}g\left(t_{j}\right). \end{array}$$ (25)

Now

$$\begin{array}{c} b_{j,n+1}=\frac{h^{\alpha }}{\alpha }\left({\left(n+1-j\right)}^{\alpha }-{\left(n-j\right)}^{\alpha }\right). \end{array}$$ (26)

The product $y_{n+1}^{p}$ is determine as,

$$\begin{array}{c} y_{n+1}^{p}=y_{0}+\frac{1}{\Gamma (\alpha )}\sum_{j=0}^{n}b_{j,n+1}f\left(t_{j},y_{j}\right). \end{array}$$ (27)

So, we can write the integral on the right-hand side an explicit calculation gives Eq (24) as,

$$\begin{array}{c} \int_{t_{0}}^{t_{n+1}}{\left(t_{n+1}-u\right)}^{\alpha -1}g_{n+1}\left(u\right)du=\sum_{j=0}^{n+1}a_{j,n+1}g\left(t_{j}\right), \end{array}$$ (28)

here,

$$\begin{array}{c} a_{j,n+1}=\{\begin{array}{cc} \frac{h^{\alpha }}{\alpha (\alpha +1)}\left(n^{\alpha +1}-\left(n-\alpha \right){\left(n+1\right)}^{\alpha }\right) & \text{if}\;j=0,\\ \frac{h^{\alpha }}{\alpha (\alpha +1)}\left({\left(n+2-j\right)}^{\alpha +1}-2{\left(n+1-j\right)}^{\alpha +1}+{\left(n-j\right)}^{\alpha +1}\right) & \text{if}\;1\leq j\leq n,\\ \frac{h^{\alpha }}{\alpha (\alpha +1)} & \text{if}\;j=n+1. \end{array} \end{array}$$ (29)

Now this gives the corrector formula, i.e., the fractional variant of one-step Adams-Moulton technique, which is

$$\begin{array}{c} y_{n+1}=y_{0}+\frac{1}{\Gamma (\alpha )}\left(\sum_{j=0}^{n}a_{j,n+1}f\left(t_{j},y_{j}\right)+a_{n+1,n+1}f\left(t_{n+1},y_{n+1}^{p}\right)\right). \end{array}$$ (30)

In order to find the value of $y_{n+1}^{p}$, we will use the predictor given in Eq (27). The complete information of the fractional version of the one step Adams-Bashforth-Moulton technique is as follows: Firstly we will calculate the predictor $y_{n+1}^{p}$ according to Eq (27), then we will find $f(t_{n+1},y_{n+1}^{p})$, then by using the predictor we will estimate the *y*_*n*+1_ by using Eq (30), and then finally we will determine *f*(*t*_*n* + 1_, *y*_*n*+1_) which is employed in next integration step. These methods are called as predictor-correct or PECE (Predict, Evaluate, Predict, Evaluate) method.

Detailed error analysis and convergence analysis for the proposed algorithm are given in [46, 47]. The technique is applicable to various real-life problems. This Predictor corrector technique is mostly used for fractional-ordered differential equations because it is easy to handle. The Adams-Bashforth Moulton technique has been successfully applied in many real-world problems. The stability of the method has already been proved in the literature [48, 49].

## Numerical simulation

The focus of this section is to validate the developed fractional WIQ model. Fractional WIQ model consists of system of coupled fractional differential equations (c.f. Eqs (1–5)). The simulated results are presented and compared with the acquired survey data of quintiles.

### Test problem 1

The simulated results are presented with the first three real-time datasets for the years 2007−2008, 2010−2011, and 2014−2015. In this section, we have used the fractional WIQ model to predict the percentage of households, in every quintile, for the year 2017−2018 using expressions (8–15). The Arithmetic Mean (A.M) of these parameter values is as follows:

*a* ≃ 0.11,
*b* ≃ 0.08,
*f* ≃ 0.07,
*s* ≃ 0.05,
*c* ≃ 0.09,
*e* ≃ 0.07,
*r* ≃ 0.06,
*h* ≃ 0.05.

**Fig 1:**
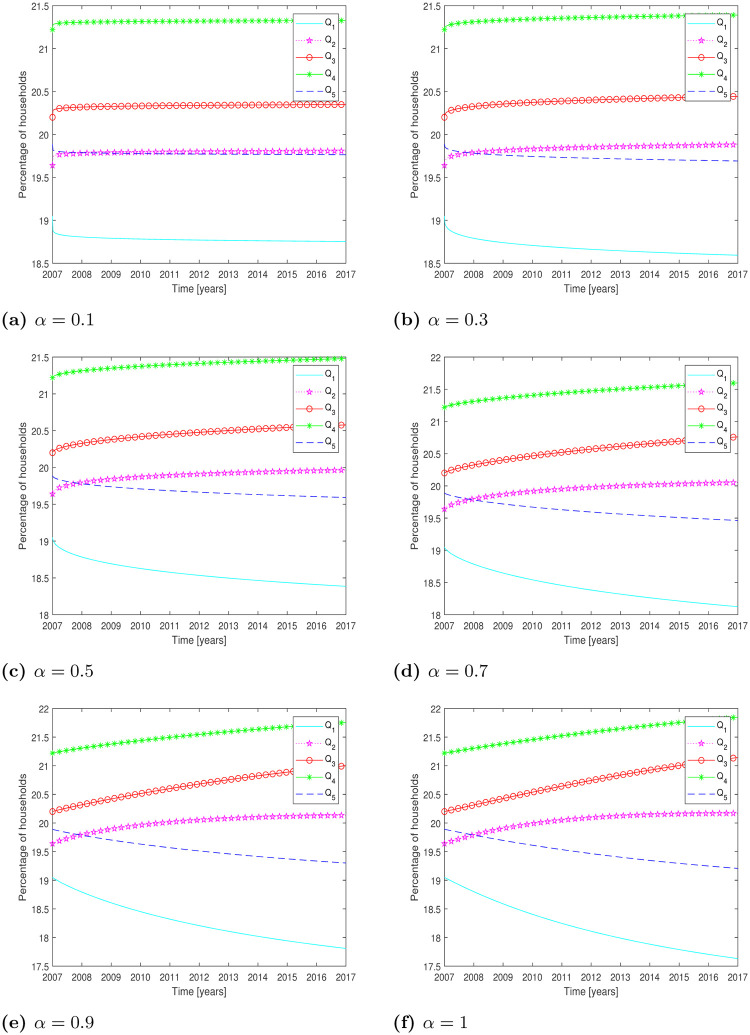
Test Problem 1: Numerical solution of the model Eqs 1–5 using Adams-Bashforth-Moulton method. **(a)** *α* = 0.1, **(b)** *α* = 0.3, **(c)** *α* = 0.5, **(d)** *α* = 0.7, **(e)** *α* = 0.9, **(f)** *α* = 1.

The simulated results are presented in Fig 1 for different values of *α*.

The corresponding quantitative results for *α* = 0.1, *α* = 0.3 and *α* = 0.5 are given in Table 1 to evaluate the difference between the exact and simulated percentage of the households. The Absolute Errors (AE) and Relative Errors (RE) are calculated by the following formula:

$$\begin{array}{c} AE=|x_{exact}-x_{simulated}|,\;\;\;RE=\frac{|x_{exact}-x_{simulated}|}{|x_{exact}|}. \end{array}$$ (31)

Errors are presented in Table 1 for *α* = 0.1, *α* = 0.3 and *α* = 0.5. Furthermore, the Table 2 presents the results for *α* = 0.7, *α* = 0.9 and *α* = 1.

**Table 2:** Numerical simulation for the year 2017 for *α* = 0.7, *α* = 0.9 and *α* = 1.

WIQ	Real	For *α* = 0.7			For *α* = 0.9			For *α* = 1		
		Simulated	AE	RE	Simulated	AE	RE	Simulated	AE	RE
*Q*_1_	19.991	18.125	1.866	0.093	17.809	2.182	0.109	17.631	2.36	0.118
*Q*_2_	20.009	20.052	0.043	0.002	20.135	0.126	0.006	20.17	0.161	0.008
*Q*_3_	19.994	20.761	0.767	0.038	21.000	1.006	0.050	21.144	1.15	0.058
*Q*_4_	19.995	21.599	1.604	0.080	21.755	1.760	0.088	21.848	1.853	0.093
*Q*_5_	20.011	19.463	0.548	0.027	19.301	0.710	0.035	19.207	0.804	0.040

CPU time for different values of *α* is presented in Table 3. The experimental order of convergence (EOC) for different values of *α* considering time steps *h* = 0.5, 1 and *h* = 1.4, 0.7 are presented in Tables 4 and 5, respectively. The numerical EOC is matched with the theoretical order of convergence [46].

**Table 3:** CPU time for different values of *α*.

Fractional order	CPU time
*α* = 0.1	0.201151
*α* = 0.3	0.201151
*α* = 0.5	0.177997
*α* = 0.7	0.208462
*α* = 0.9	0.206175
*α* = 1	0.223060

**Table 4:** EOC using *h* = 1 and *h* = 0.5 for different values of *α*.

	*α* = 0.1	*α* = 0.3	*α* = 0.5	*α* = 0.7	*α* = 0.9	*α* = 1
*Q*_1_	1.19	1.32	1.35	1.36	1.36	1.36
*Q*_2_	1.22	1.25	1.26	1.26	1.22	1.18
*Q*_3_	1.30	1.39	1.40	1.40	1.40	1.40
*Q*_4_	1.19	1.35	1.37	1.38	1.38	1.38
*Q*_5_	1.30	1.35	1.38	1.38	1.38	1.38

**Table 5:** EOC using *h* = 1.4 and *h* = 0.7 for different values of *α*.

	*α* = 0.1	*α* = 0.3	*α* = 0.5	*α* = 0.7	*α* = 0.9	*α* = 1
*Q*_1_	1.02	1.19	1.21	1.21	1.21	1.21
*Q*_2_	1.07	1.12	1.13	1.12	1.08	1.07
*Q*_3_	1.21	1.25	1.26	1.26	1.25	1.25
*Q*_4_	1.19	1.21	1.23	1.23	1.24	1.24
*Q*_5_	1.19	1.22	1.23	1.24	1.23	1.23

For integer order (i.e *α* = 1) the results show more Relative Errors (RE) and Absolute Errors (AE) than the other values of *α*. From Tables 1 and 2, we can see that the results improve for the smaller values of *α* as *α* = 0.1 and *α* = 0.3. The simulated results are consistent with the real data of the percentage of households for *α* = 0.1, as compared to other values. The absolute and relative errors show that the simulated data and the real data are closer to each other for *α* = 0.1. Furthermore, the reasonable range of *α* for the considered problem is less than 0.5. Graphically, the simulated results of quintiles for different fractional order is presented in Fig 1. From the figures, one can see the deviation of quintiles from the year 2007 to 2017.

The results indicate that, in every quintile, real and simulated household percentages are closer to each other. Furthermore, the relative error is less than 1% which demonstrates the accuracy of the established model. On the basis of these results, the importance of FC is shown.

### Test problem 2

After validating the accuracy of our established fractional WIQ model, we have employed it to predict the household percentages for the year 2020 by using *α* = 0.1. In this problem, we have acquired the parametric values considering the data from the year 2007 to 2017.

The average values of the parameters are as follows:

*a* ≃ 0.11,
*b* ≃ 0.08,
*f* ≃ 0.07,
*s* ≃ 0.05,
*c* ≃ 0.09,
*e* ≃ 0.07,
*r* ≃ 0.06,
*h* ≃ 0.05.

**Fig 2:**
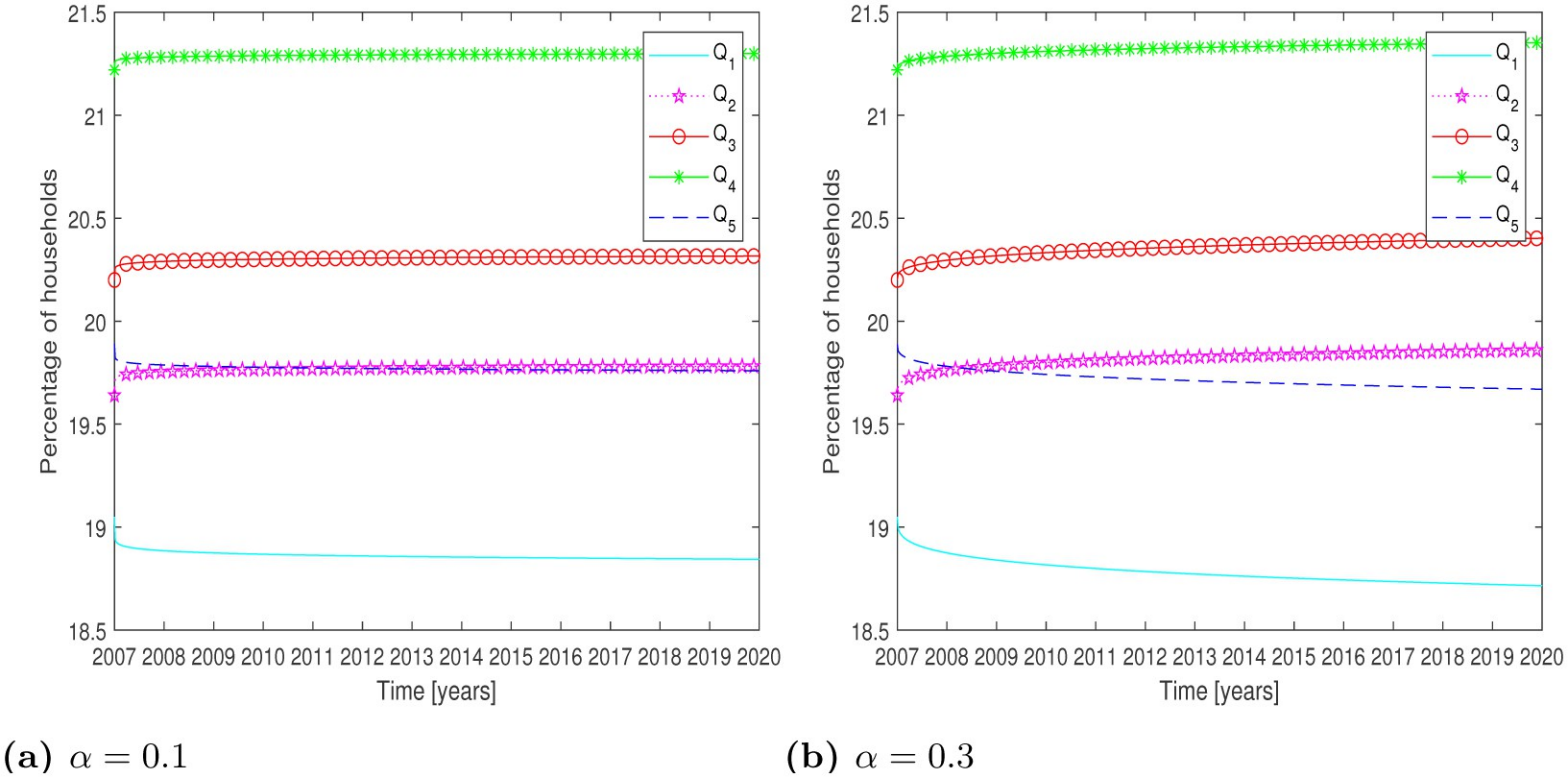
Test Problem 2: Numerical solution of the model Eqs 1–5, presenting prediction the year 2020. **(a)** *α* = 0.1 **(b)** *α* = 0.3.

The corresponding simulation results for the year 2007-2020 have shown in Fig 2:

In Table 6, the household percentage is shown for the year 2020.

**Table 6:** Numerical simulation for the year 2020 when *α* = 0.1 and *α* = 0.3.

WIQ	Simulated (%age) for *α* = 0.1	Simulated (%age) for *α* = 0.3
*Q*_1_	18.845	18.715
*Q*_2_	19.781	19.860
*Q*_3_	20.316	20.403
*Q*_4_	21.299	21.353
*Q*_5_	19.759	19.669

### Parametric study of fractional WIQ model

In this test problem, the parametric study of the fractional WIQ model is presented. The purpose of this section is to validate/analyze the fractional WIQ model by varying different parameters. The variation of the parameters is an evaluation of the model to identify the impact of parameters on households. The influence of these parameters on households is discussed by changing one parameter while keeping all other parameters constant. The value of the parameter is increased or decreased by 25% from its original value given in test problem 1.

**Fig 3:**
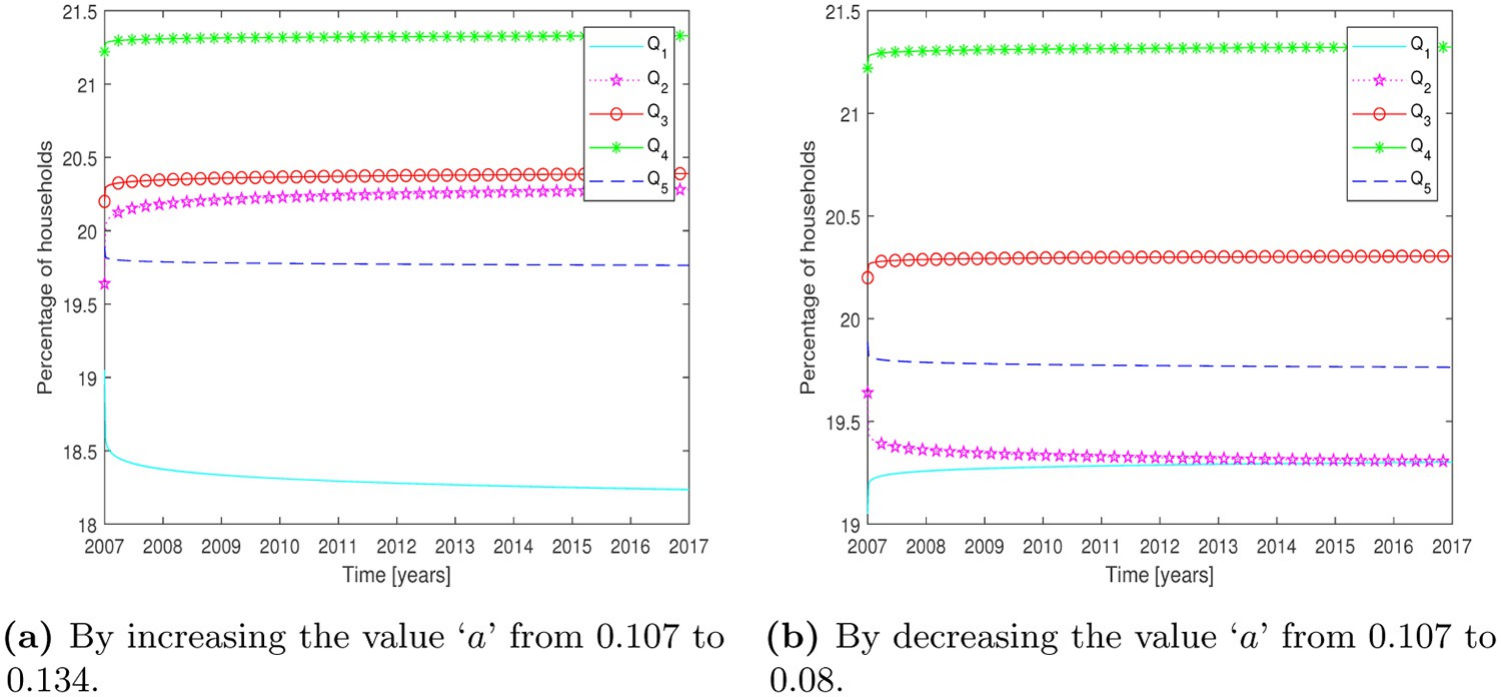
Effect of parameter *a* on the model equations by increasing and decreasing value of *a* to 25%. **(a)** By increasing the value ‘*a*’ from 0.107 to 0.134. **(b)** By decreasing the value ‘*a*’ from 0.107 to 0.08.

The initial conditions and parameter values, given in test Problem 1, are used. Fig 3a & 3b show the influence of parameter ‘*a*’on quintiles. Increasing parameter ‘*a*’increase household percentage in *Q*_2_ while decrease the percentage of *Q*_1_ (Fig 3a). Similarly, decreasing parameter ‘*a*’brings increase in *Q*_1_ and decrease in *Q*_2_ (Fig 3b). The results are synchronized with the model equations which verifies the correctness of the model formulations. In a similar manner, the effects of different parameters can be studied and omitted here due to repetition.

## Conclusion

The purpose of this study was to develop a coherent mathematical model to forecast the wealth indices of the households of Punjab, Pakistan. The wealth index is important to measure the economic and social well-being of households. It is imperative to examine the inequalities for the excess of the ownership of assets between the rich and poor. The wealth index assigned a score to the households and then divides the population into equal segments that define wealth quintiles as the lowest, second, middle, fourth, and highest. The fractional WIQ model was developed using the system of coupled fractional differential equations. The model equations were solved numerically with the help of the Adams-Bashforth-Moulton method and the stability of the model was discussed. The numerical results for the different values of *α* were calculated. The results for lessor values of *α*, such as *α* = 0.1 and *α* = 0.3 were better as compared to *α* = 1.

The simulated results were validated with real-time data. To quantify the difference between real data and the simulated percentage of households, absolute and relative errors were calculated. The relative error is less than 1% in all considered problems verifying that the fractional WIQ model has been formulated correctly. Systematic parametric studies were carried out to demonstrate the influence of the variation of parameters on the fractional WIQ model. This study enlightens the importance of FC in different fields, especially in economics.

The developed fractional WIQ model can be used to forecast the households’ socioeconomic conditions in the population. In a similar manner, different models can be formed considering different surveys such as the availability of water facilities to the population and the impact of viral disease on the population, etc. Moreover, future results obtained using the fractional WIQ model can help planning personnel & policymakers counter threats to a segment of the population. However, the results obtained cannot be used for other regions as the WIQs are a relative measure, and ownership of assets is different in different regions. It is utterly possible that the highest quintile of our dataset represents a lower quintile for the other region.

This study is an effort to provide more deep insights into the variation of the quintiles to improve the households’ socioeconomic condition. This work can be used to forecast different aspects of well-being that can cause huge unrest among a particular quintile at a certain time. Furthermore, this model can be utilized to program different social welfare schemes for the betterment of society such as the Benazir Income Support Program and Ehsaas Kafalat Program for the poor and the educational Ehsaas scholarship for the poor and middle class. The model also provides the opportunity for the researchers to use WIQs of their respective regions to forecast the households’ socioeconomic conditions.
